# Liver-Specific Overexpression of Prostasin Attenuates High-Fat Diet-Induced Metabolic Dysregulation in Mice

**DOI:** 10.3390/ijms22158314

**Published:** 2021-08-02

**Authors:** Tetsuo Sekine, Soichi Takizawa, Kohei Uchimura, Asako Miyazaki, Kyoichiro Tsuchiya

**Affiliations:** 1 Interdisciplinary Graduate School of Medicine and Engineering, University of Yamanashi, Chuo 4093898, Japan; tsekine@yamanashi.ac.jp (T.S.); stakizawa.ych@gmail.com (S.T.); kuchimura@yamanashi.ac.jp (K.U.); 2 Internal Medicine, Yamanashi Prefectural Central Hospital, Kofu 4008506, Japan; 3 Isawa Hot Spring Hospital, Fuefuki 4060023, Japan; atiatiasasuke@yahoo.co.jp

**Keywords:** diabetes mellitus, extracellular signal-regulated MAP kinase, fatty liver, matrix metalloproteinase 14, serine protease

## Abstract

The liver has a most indispensable role in glucose and lipid metabolism where we see some of the most serious worldwide health problems. The serine protease prostasin (PRSS8) cleaves toll-like receptor 4 (TLR4) and regulates hepatic insulin sensitivity under PRSS8 knockout condition. However, liver substrate proteins of PRSS8 other than TLR4 and the effect to glucose and lipid metabolism remain unclarified with hepatic elevation of PRSS8 expression. Here we show that high-fat-diet-fed liver-specific PRSS8 transgenic mice improved glucose tolerance and hepatic steatosis independent of body weight. PRSS8 amplified extracellular signal-regulated kinase phosphorylation associated with matrix metalloproteinase 14 activation in vivo and in vitro. Moreover, in humans, serum PRSS8 levels reduced more in type 2 diabetes mellitus (T2DM) patients than healthy controls and were lower in T2DM patients with increased maximum carotid artery intima media thickness (>1.1 mm). These results identify the regulatory mechanisms of PRSS8 overexpression over glucose and lipid metabolism, as well as excessive hepatic fat storage.

## 1. Introduction

During the last decades, the prevalence of obesity reached proportions of a serious epidemic, and impaired glucose and lipid metabolism propitiated by this disease is one of the major health problems worldwide. In this scenario, the liver has a most indispensable role in glucose and lipid metabolism that is affected, especially by prolonged obesity, with fat accumulating in the liver and insulin resistance being prone. On another hand, prostasin (PRSS8) is a serine protease located at the cell membrane and was firstly purified from seminal fluid, and now, PRSS8 has been found to be expressed in several tissues such as the liver [1]. Although PRSS8 cleaves proteins that are thereby activated or inactivated due to the arising conformational changes, the role of PRSS8 in many organs remains largely unexplored. In the kidney, PRSS8 cleaves and activates epithelial sodium channel (ENaC) in collecting ducts of nephron [2]. The gain-of-function mutation of ENaC is associated with Liddle syndrome, an inherited form of hypertension. In the liver, a previous study encompassing liver-specific PRSS8 knockout (LKO) mice evidences PRSS8 as a suppressor of liver inflammation triggered by lipopolysaccharide or saturated fatty acid, for this cleaving and deactivating toll-like receptor (TLR) 4 [1]. TLR4 is a widely known mediator of inflammatory response in adipose tissue, skeletal muscle, islets, and liver [3,4,5], thereby at least in the last case contributing to insulin resistance. Additionally, the PRSS8 levels are diminished by the endoplasmic reticulum stress, a characteristic condition with high-fat diet or obesity, leading to insulin resistance when TLR4 was less inactivated [1].

Thus, prior research suggests heightened PRSS8 levels in the liver as a considerable pharmacological target to appease insulin resistance in situations of impaired glucose metabolism therapy. However, liver substrate proteins of PRSS8 other than TLR4, in this context, remain unclarified, as well as the association between fatty liver or deficient lipid metabolism, brought about by obesity, and PRSS8. Being so, the precise determination of the effect of a given PRSS8 increase in the liver over glucose and lipid metabolism is essential. Therefore, the aim of our work was further unveiling of this new protein-associated mechanism of mediating glucose and lipid metabolism, with hepatic elevation of PRSS8 expression.

## 2. Results

### 2.1. HFD-Fed LTg Mice Evidenced an Improved Glucose Tolerance Independent of Body Weight

**Figure 1:**
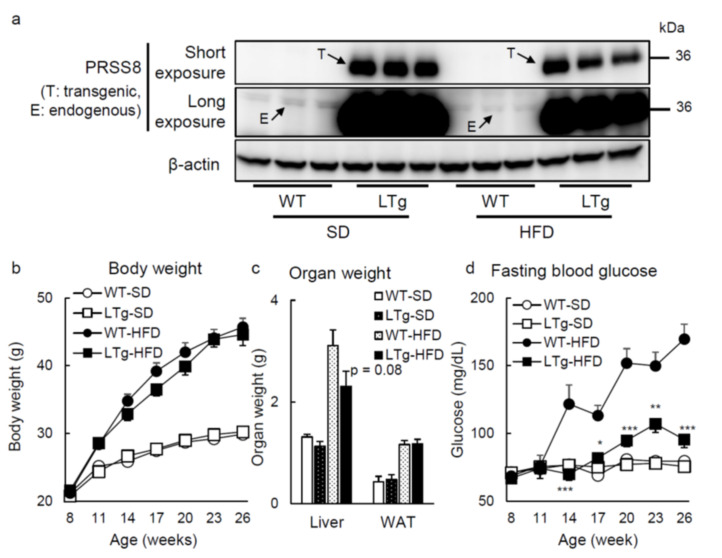
LTg mice under feeding HFD improved fasting blood glucose levels. (**a**) Western blotting analysis of PRSS8 in the liver tissues of the WT/LTg mice under overnight fasting condition (14 h) and feeding SD or HFD. Representative Western blot images after short and long exposure were shown (n = 3 mice per group). (**b**–**d**) Body (BW) and organ (liver and epididymal white adipose tissues [WAT]) weight, and fasting blood glucose (FBG) levels were shown in WT/LTg mice under feeding SD or HFD (n = 10–17 mice per group). Values are shown as the mean ± SEM; * *p*-value < 0.05, ** *p*-value < 0.01, and *** *p*-value < 0.001. The statistical analysis was used two-way repeated measures analysis of variances for BW and FBG in SD or HFD, or *t* test for organ weight in HFD.

**Figure 2:**
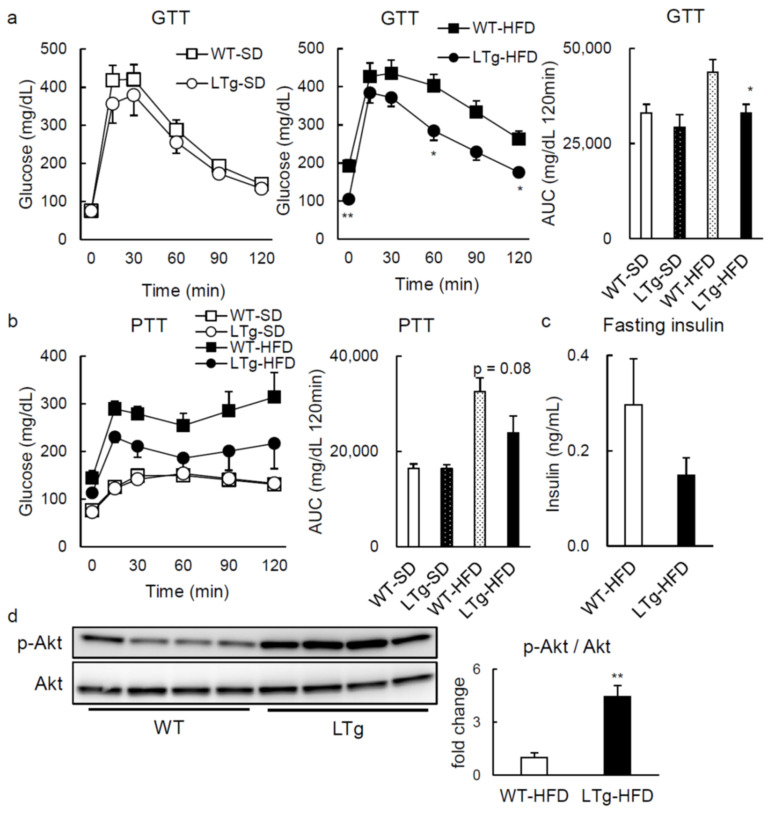
LTg mice under feeding HFD improved insulin resistance. (**a**) Blood glucose levels during glucose tolerance test (GTT) (2 g/kg for SD and 1 g/kg for HFD) and the total area under the curve (AUC) in WT/LTg mice under feeding SD or HFD after overnight fast (14 h) (n = 8–11 mice per group). Values are shown as the mean ± SEM; * *p*-value < 0.05 and ** *p*-value < 0.01. Two-way repeated measures analysis of variances was used for GTT in SD or HFD, and *t* test was used for AUC in HFD. (**b**) Pyruvate tolerance test (PTT) (2 g/kg) and the total AUC in WT/LTg mice under feeding SD or HFD after overnight fast (14 h) (n = 7–11 mice per group). Values are shown as the mean ± SEM. Two-way repeated measures analysis of variances was used for PTT in SD or HFD, and *t* test was used for AUC in HFD. (**c**) Blood insulin levels in HFD mice after overnight fast (14 h) (n = 4 mice per group). Values are shown as the mean ± SEM. (**d**) Western blotting and quantification of phosphorylated and total Akt in the liver of the WT/LTg mice under HFD feeding after overnight fast (14 h) (n = 4 per group). Values are shown as the mean ± SEM; ** *p*-value < 0.01 (*t* test).

With the purpose of assessing metabolic phenotypes with an increase in hepatic PRSS8 levels, we generated liver-specific PRSS8 transgenic (LTg) mice. Western blotting analysis confirmed the overexpression of PRSS8 in the livers of LTg mice after 14-h overnight fast (Figure 1a): endogenous and transgenic PRSS8 bands were detected at 40 kDa after long exposure, and 36 kDa, respectively. The body weight (BW) was not affected by PRSS8 overexpression when mice were fed, interestingly, with both standard diet (SD) and 60 kcal% high-fat diet (HFD) (Figure 1b). The weight of the liver and the epididymal white adipose tissues (WAT) was not significantly different between SD-fed LTg and WT mice. In contrast, there was a trend of decrease in liver weight of HFD-fed LTg mice compared to WT mice (*p* = 0.08) (Figure 1c). Coherently with this, fasting blood glucose levels after 6 weeks with HFD were significantly lower in LTg mice than those in WT ones (Figure 1d). In opposition to glucose tolerance test (GTT) after 22–25 weeks of SD feeding showing no disparity when WT and LTg mice are paralleled, HFD-fed LTg mice displayed better glucose tolerance compared to HFD-fed WT mice (Figure 2a). Pyruvate tolerance test (PTT) also substantiated that glucose production tends to be hindered in HFD-fed LTg mice, in contrast with HFD-fed WT (Figure 2b). The fasting insulin levels were comparable between WT and LTg mice, whereas fasting glucose levels were significantly lower in LTg mice than those in WT mice (Figure 2c). Fasting liver Akt phosphorylation was enhanced in LTg mice, compared to WT mice, when encompassing the HFD (Figure 2d).

Altogether, our observations suggest that hepatic overexpression of PRSS8 protects mice from HFD-prompted glucose intolerance, in a manner independent of their BW.

### 2.2. HFD-Fed LTg Mice Showed Improved Hepatic Steatosis

**Figure 3:**
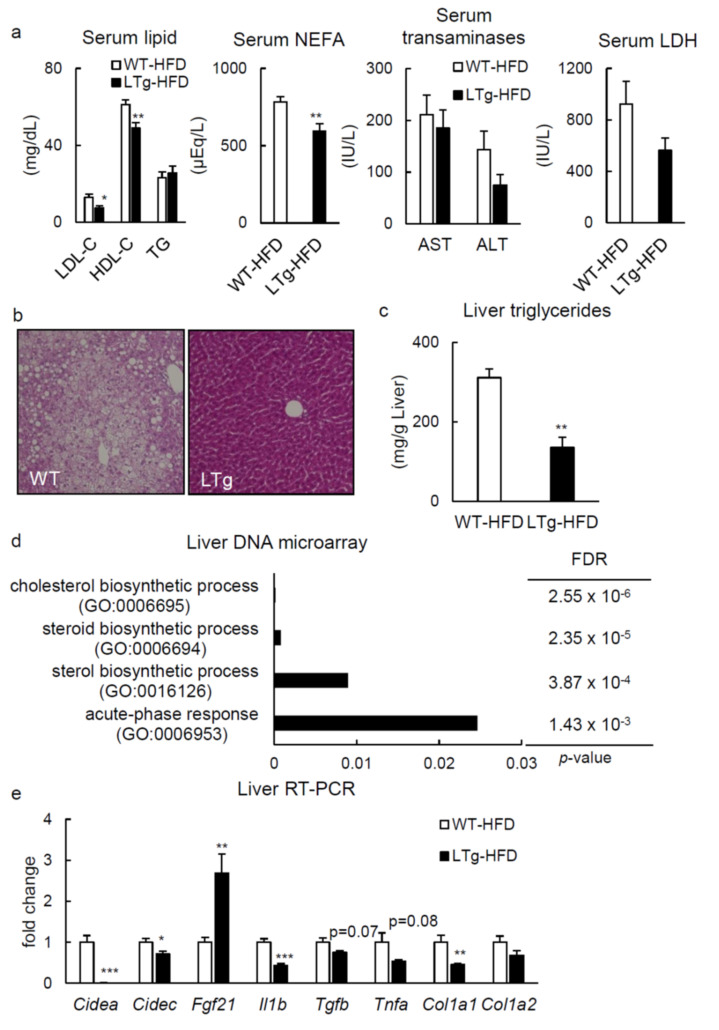
LTg mice under feeding HFD improved serum cholesterol levels and fatty liver. (**a**) Serum low-(LDL-C) and high-density lipoprotein cholesterol (HDL-C), triglyceride (TG), non-esterified fatty acid (NEFA), aspartate (AST) and alanine aminotransferase (ALT), and lactate dehydrogenase (LDH) levels in HFD-fed mice after overnight fast (14 h) (8–9 mice per group). Values are shown as the mean ± SEM; * *p*-value < 0.05 and ** *p*-value < 0.01 (*t* test). (**b**) Hematoxylin eosin stains of representative liver samples derived from a WT/LTg mouse under HFD condition (×400). (**c**) Hepatic triglyceride levels in HFD mice after overnight fast (14 h) (n = 7–8 mice per group). Values are shown as the mean ± SEM; ** *p*-value < 0.01 (*t* test). (**d**) The ontology terms enriched among the downregulated (<0.67-fold) mRNAs liver from a LTg mouse compared those from a WT mouse under feeding HFD after overnight fast (14 h). False discovery rates (FDR) are shown by Benjamini and Hochberg. (**e**) RT-PCR of lipid metabolism, inflammatory, and fibrosis genes in the liver tissue of the WT/LTg mice under feeding HFD after overnight fast (14 h) (n = 5–7 per group). Values are shown as the mean ± SEM; * *p*-value < 0.05, ** *p*-value < 0.01, and *** *p*-value < 0.001 (*t* test).

LTg mice fed with HFD after 14-h fast presented lower serum low- (LDL) and high-density lipoprotein (HDL) cholesterol and non-esterified fatty acid (NEFA) concentrations than WT animals (Figure 3a). In HFD-fed LTg mice, hepatic steatosis was attenuated compared to WT mice (Figure 3b,c). In contrast, notwithstanding, no significant dissimilarity was noted in serum LDL- and HDL-cholesterol, aspartate, alanine aminotransferase, and lactate dehydrogenase proportions, between SD-fed LTg and WT mice (Supplementary Figure S1). Furthermore, pathway analysis of the liver microarray data revealed a substantial alteration for genes connected with cholesterol, steroid, and sterol biosynthetic processes in HFD-fed LTg mice, as compared to the WT (Figure 3d). In quantitative real-time polymerase chain reaction (RT-PCR) analysis, a significant downregulated expression was demonstrated for *Cidea*, *Cidec*, *Il1b*, and *Col1a1* genes in liver from LTg mice with a HFD, parallel to those of the WT (Figure 3e). Additionally, hepatic expression rate of *Fgf21* was clearly heightened in HFD-fed mice, contrary to that in WT (Figure 3e). Moreover, synthesis of cluster of differentiation 36 (CD36) protein in the liver, which presumably lessened HFD-triggered hepatic steatosis in mice [6], was suppressed both in terms of protein (Supplementary Figure S2a) and respective coding mRNA (Supplementary Figure S2b), when LTg mice are compared to WT mice.

Globally, hepatic overexpression of PRSS8 was pointed as an attenuator against HFD-driven hepatic lipid accumulation, apparently accompanying this with a modification of a serum lipid profile and hepatic CD36 expression.

### 2.3. PRSS8 Amplified ERK Phosphorylation Associated with MMP14 Activation in HepG2 Cells

**Figure 4:**
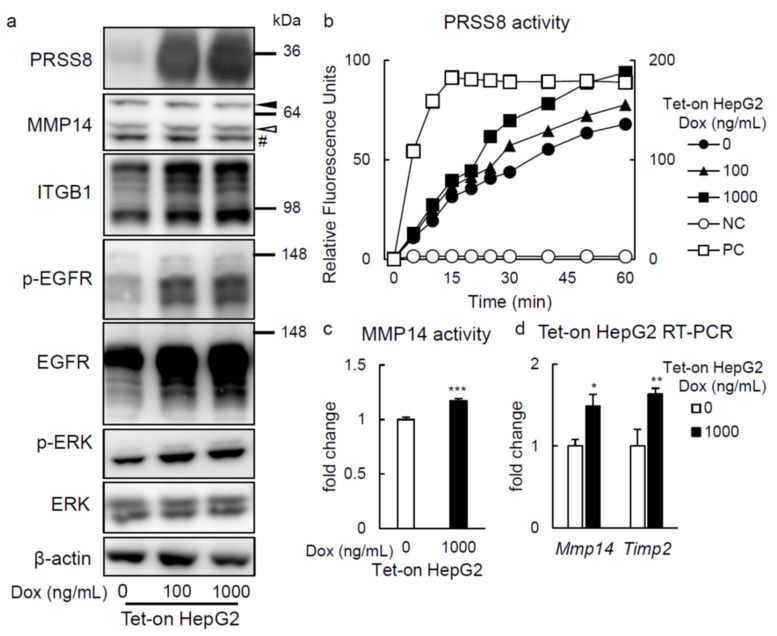
Tet-on HepG2 cells were generated and over expression of PRSS8 activated MMP14 cascades. (**a**) Representative Western blot of three independent experiments for PRSS8 and matrix metalloproteinase (MMP) 14 cascade in the Tet-on HepG2 cells under 72 h exposure to each concentration of doxycycline (Dox) conditions. A solid arrow head; pro-MMP14, an open arrow head; activated MMP14, #; nonspecific bands. (**b**) Representative PRSS8 activity measured by fluorogenic substrate (Acetyl-Lys-His-Tyr-Arg-α-[4-methylcoumaryl-7-amide]) of four independent experiments in the Tet-on HepG2 cells. The negative control (NC; T-PER Tissue Protein Extraction Reagent) and positive control (PC; trypsin 1 μg) were located. The right vertical axis is for PC and the left vertical axis for all others. (**c**) The MMP14 activity of the Tet-on HepG2 cells was measured (n = 4 per group). Values are shown as the mean ± SEM; *** *p*-value < 0.001 (*t* test). (**d**) RT-PCR of MMP14 and tissue inhibitor of metalloprotease (TIMP) 2 genes in the liver of the Tet-on HepG2 cells under 72 h exposure to each concentration of Dox conditions (n = 4–6 per group). Values are shown as the mean ± SEM; * *p*-value < 0.05 and ** *p*-value < 0.01 (*t* test).

Aiming to explore the mechanism by which PRSS8 overexpression improves HFD-induced glucose intolerance and hepatic, we established cell lines using a doxycycline (Dox)-inducible gene expression system in HepG2 cells (Tet-on HepG2). In such cells, treatment with Dox enlarged PRSS8 mRNA (Supplementary Figure S3a) and protein (Figure 4a) expression, as well as its activity (Figure 4b). Initially, we studied TLR4 expression, which was reported to be cleaved and inhibited by PRSS8 in forecited LKO mice. Nonetheless, the expression of TLR4 showed no alteration neither in vitro nor in vivo (Supplementary Figure S4a,b). Posteriorly, as a candidate molecule linking PRSS8 to glucose metabolism and hepatic lipid accumulation, we focused on the matrix metalloproteinase 14 (MMP14; also known as MT1-MMP). MMP14 is a type 1 membrane protein, with single transmembrane and extracellular catalytic domains. MMP14 is generated as zymogen, pro-MMP14, and activated by furin-dependent and -independent proteolytic cleavage to become activated MMP14, while it is inhibited by tissue inhibitor of metalloproteinases 2 (TIMP2), assembling with it a stable complex [7,8]. Whereas MMP14 expression was unaffected by overproduced PRSS8 in the Tet-on HepG2 cells (Figure 4a), the activity of this protein was significantly stimulated by the former condition (Figure 4c), similarly to *Timp2* mRNA synthesis (Figure 4d). Although representative genes related to glucose and lipid metabolism were not significantly affected by heightened PRSS8 concentration in Tet-on HepG2 cells (Supplementary Figure S3a,b), CD36 protein expression was reduced in the Tet-on HepG2 cells (Supplementary Figure S3c).

Extracellular signal-regulated kinases (ERKs) play crucial roles in hepatic glucose and lipid metabolisms, in line with when ERK1 knockout mice displayed excess of weight and insulin resistance, besides liver-specific ERK2-knockout mice presenting also hepatic steatosis, glucose intolerance and reduced insulin sensitivity [9,10]. MMP14 seemingly phosphorylates ERK via Integrin subunit β1 (ITGB1) in mammary epithelial cells [11]. Aside from this, it has been reported that in epidermal growth factor receptor (EGFR) signaling, this protein is phosphorylated by PRSS8, thereby activating ERK, via phosphorylation too, in hepatocytes [12,13]. As expected, in the Tet-on HepG2 cells an augmented expression of PRSS8 led to that of ITGB1 protein, concomitantly with raised levels of EGFR and ERK phosphorylation (Figure 4a).

In summary, the later results underline that PRSS8 appears to contribute to MMP14 activation in Tet-on HepG2 cells, here being hypothesized that PRSS8 activates ERK in a MMP14- and EGFR-mediated manner.

### 2.4. ERK Phosphorylation Is Elevated in Liver of HFD-fed LTg Mice, Associated with an Increased ITGB1 Expression and MMP14 Activity

**Figure 5:**
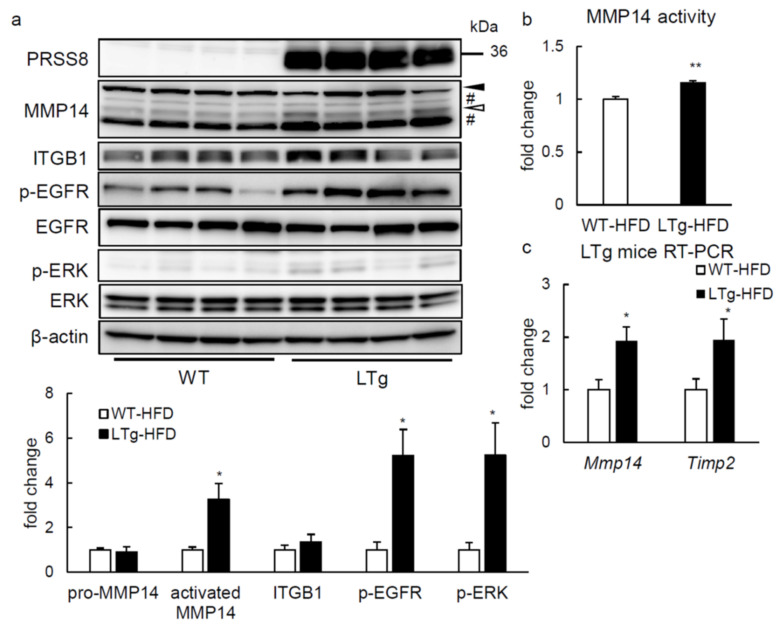
LTg mice activated MMP14 cascades similar to the Tet-on HepG2 cells. (**a**) Western blotting analysis and quantification of matrix metalloproteinase (MMP) 14 cascade in the liver of the WT/LTg mice under feeding HFD after overnight fast (14 h) (n = 4 per group). A solid arrowhead; pro-MMP14, an open arrowhead; activated MMP14, #; nonspecific bands. Phospho-EGFR and -ERK were normalized to total EGFR and ERK, respectively. Other proteins were normalized to β-actin. Values are shown as the mean ± SEM; * *p*-value < 0.05 (*t* test). (**b**) The MMP14 activity of the liver of WT/LTg mice after overnight fast (14 h) was measured (n = 4 per group). Values are shown as the mean ± SEM; ** *p*-value < 0.01 (*t* test). (**c**) RT-PCR of MMP14 and tissue inhibitor of metalloprotease (TIMP) 2 genes in the liver of the WT/LTg mice under feeding HFD after overnight fast (14 h) (n = 5–6 per group). Values are shown as the mean ± SEM; * *p*-value < 0.05 (*t* test).

**Figure 6:**
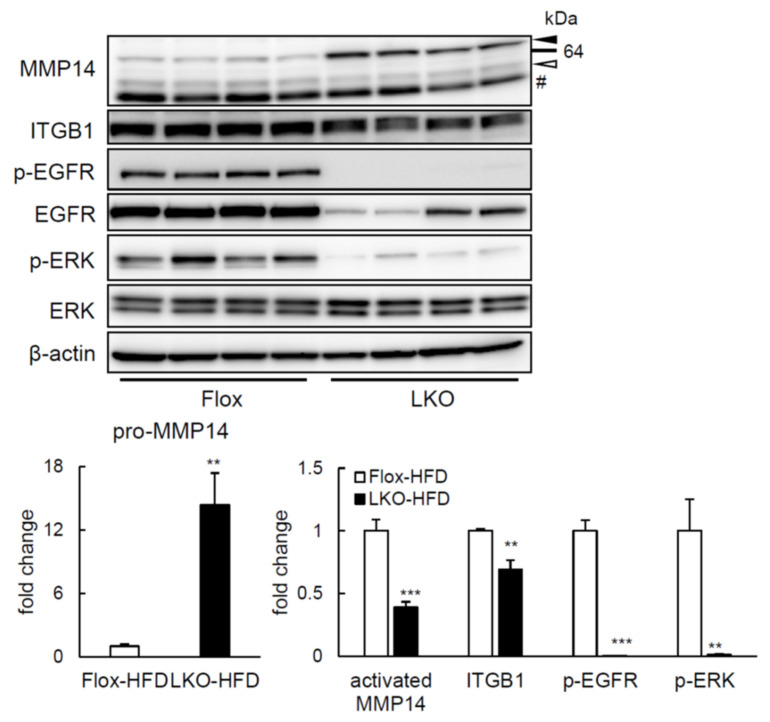
LKO mice inactivated MMP14 cascades. Western blotting analysis and quantification of MMP14 cascade in the Flox/LKO mice under feeding HFD after overnight fast (14 h) (n = 4 per group). A solid arrow head; pro-MMP14, an open arrow head; activated MMP14, #; nonspecific bands. Phospho-EGFR and -ERK were normalized to total EGFR and ERK, respectively. Other proteins were normalized to β-actin. Values are shown as the mean ± SEM; ** *p*-value < 0.01 and *** *p*-value < 0.001 (*t* test).

As observed in Tet-on HepG2 cells, whereas pro-MMP14 production did not vary in the liver of HFD-fed LTg mice after overnight fast (14 h), compared to WT animals (Figure 5a), protein amount of activated MMP14 and MMP14 activity were considerably enhanced (Figure 5a,b). In agreement with it, *Mmp14* and *Timp2* gene expression was also greater in liver of HFD-fed LTg mice after overnight fast (14 h) (Figure 5c). Moreover, phosphorylation of EGFR and ERK was also increased in HFD-fed LTg mice (Figure 5a). Unlike Tet-on HepG2 cells, hepatic ITGB1 expression was comparable between HFD-fed LTg and WT mice. In contrast, liver-specific PRSS8-knockout mice subject to HFD presented reduced activated MMP14 and ITGB1 protein levels, and phosphorylation of ERK and EGFR in the liver, contrary to PRSS8^lox/lox^ (Flox) mice (Figure 6). Concluding, these data suggest that PRSS8-induced ERK phosphorylation may add to an improved glucose tolerance and simultaneous appeased hepatic lipid accumulation on HFD-fed LTg mice, feasibly by means of the MMP14 induction and/or EGFR phosphorylation.

### 2.5. Serum Soluble PRSS8 Concentration Is Superior in HFD-Fed LTg Mice, Although Being Lessened in Patients with Type 2 Diabetes

Because soluble PRSS8 can be detected in the serum [1], we then assessed whether liver-specific PRSS8 overexpression correlated to the increase of serum PRSS8 concentration. This was markedly intense in LTg mice, when compared to WT mice after overnight fast (Supplementary Figure S5a). In the WAT of HFD-fed LTg mice after overnight fast, *Glut4*, *Acox1*, and *Ppara* expression was significantly larger than that of WT mice (Supplementary Figure S5b). On the other hand, expression of *Tnfa*, *Ccl2*, *Ccr2*, and *Emr1* genes was attenuated in WAT of HFD-fed LTg mice compared to that of WT mice.

**Figure 7:**
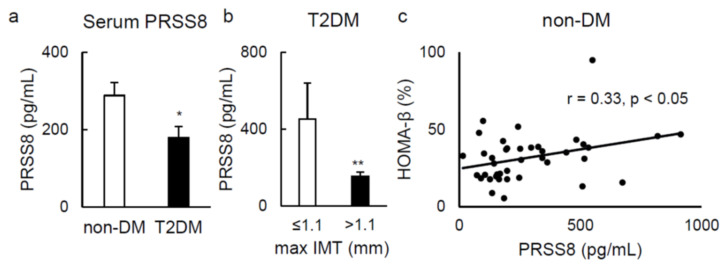
Serum PRSS8 levels was negatively associated with the presence of type 2 diabetes and increased intima media thickness. (**a**) The ELISA of human PRSS8 levels in the serum of the nondiabetes mellitus (non-DM) and type 2 diabetes mellitus (T2DM) participants (n = 39–40 per group). Values are shown as the mean ± SEM; * *p*-value < 0.05 (*t* test). (**b**) The maximum intima media thickness (max IMT) measured by carotid ultrasonography in T2DM. Values are shown as the mean ± SEM; ** *p*-value < 0.01 (*t* test). (**c**) Homeostatic model assessment beta cell function (HOMA-β) in non-DM was shown (Pearson product-moment correlation coefficient).

On a distinct note, in humans, serum PRSS8 concentration was evidently inferior in patients with type 2 diabetes than that in healthy ones (Figure 7a). Moreover, serum PRSS8 concentration was also notably lower in patients with increased maximum carotid artery intima media thickness (max IMT) (>1.1 mm), if paralleled to patients with normal parameter (≤1.1 mm) (Figure 7b). Serum levels of PRSS8 were associated with an index of insulin secretory function examined by homeostatic model assessment of β-cell function (HOMA-β) in nondiabetic individuals (Figure 7c).

Serum PRSS8 results support, collectively, that an improved expression of liver PRSS8 culminates in elevation of this form of PRSS8, which may be involved in the pathophysiology of type 2 diabetes and diabetes-associated atherosclerosis as well.

## 3. Discussion

The global prevalence of hyperglycemia and hyperlipidemia causing obesity is one of the current most imperative health problems. In the cited former study using the LKO mice fed HFD, liver-specific ablation of PRSS8 promoted insulin resistance driven by presence of a HFD [1]. However, considered a pharmacological purpose point of view, the use of PRSS8 for obesity-induced insulin resistance yet implies the elucidation of whether increase in PRSS8 improves insulin resistance. Our present work evidences the potential therapeutic effects of an increase of hepatic PRSS8 levels over obesity-triggered insulin resistance and dyslipidemia.

In the LKO mice, a loss-of-function mouse model of hepatic PRSS8 was attained, proving that physiologically expressed PRSS8 releases the ectodomain of TLR4 by cleaving it, which ultimately results in a reduction of full-length form and TLR4 activation [1]. Moreover, restoring PRSS8 hepatic synthesis of the LKO mice decreased TLR4 expression and ameliorated insulin resistance with the HFD. However, unlike LKO mice, hepatic TLR4 expression was not altered in LTg mice compared to WT mice. We assume that the supraphysiological expression of PRSS8 does not cleave TLR4 more than the physiological level, possibly because of the amount/activity balance of protease and substrate. Indeed, basal hepatic inflammation levels as assessed by inflammatory gene expression showed little difference between LTg and WT mice fed a HFD. Although we did not directly assess the hepatic response to endotoxin, we consider that LTg mice are unlikely to be protected from endotoxemia and/or basal inflammation related to HFD.

Accordingly with previous research, PRSS8 is known to promote EGFR phosphorylation [12], and the phosphorylation of EGFR leads the phosphorylation of ERK [14,15,16]. The latter is one of the mitogen-activated protein kinases (MAPKs), these playing a prominent part in processes that regulate liver metabolism [17,18,19]. In this set, C-Jun N-terminal kinase and p38 MAPK are both stimulated by stress signals and appear as crucial effectors among physiological and pathophysiological hepatic metabolism [20,21]. Coherently with such fact, recent studies addressed ERK1 knockout mice having a weight gain and insulin resistance, while ERK2 liver-specific knockout mice also developed hepatic steatosis, glucose intolerance, and diminished insulin sensitivity [9,10]. Additionally, adenovirus-mediated induction of MAPK kinase 1, the upstream regulator of ERK, notoriously relieved liver steatosis in leptin receptor-deficient mice [19]. Here having noticed an increase in EGFR and ERK phosphorylation in both Tet-on HepG2 cells and LTg mice, whereas it decreased of one in LKO mice beforehand, the phenotypes of LTg and LKO mice seem consistent with those above described of ERK knockout and activation mice.

Inclusively, we underlined that overexpression of PRSS8 prompted higher MMP14 activity in not only Tet-on HepG2 cells but also LTg mice liver. As a matter of fact, MMP14 is being considered in areas such as tumor invasion of several organs [22,23,24]. On the other hand, there are few reports on the role of MMP14 in glucose and lipid metabolism, in spite of having been reported to contribute to the improvement of liver fibrosis [25]. Furthermore, MMP14 is synthesized as a zymogen, prone to be processed to its mature, catalytically active form, activated MMP14, after the removal of respective regulatory propeptide domain. One of the proteases responsible for this is furin [7], which recognizes the sequence of Arg-Arg-Lys-Arg in MMP14, and activates this protein. Based on a distinct study, MMP14 was activated by other proteases, notwithstanding which exact proteases being unclear [8]. Furin and PRSS8 belong to the same serine protease family, besides the sequence of furin cleavage to MMP14 activates being equally recognized by PRSS8 [26]. In addition, MMP14 activates ERK [27], as stated, consequently, MMP14 entering a cross-talk in the PRSS8-EGFR-ERK axis, although the total function of MMP14 amid glucose and lipid metabolism is not completely clarified.

Still regarding MMP14, it seems also to be involved in directly activating MMP2 [28] and indirectly activating MMP13 [29], at the same time that it regulates proteins out of the MMP family, such as ITGB1, CD44, and platelet-derived growth factor receptor [11,27,30]. Previous evidence highlights an essential role of ITGB1 in liver regeneration and an important cross-talk between ITGB1 and insulin influence over skeletal muscle [27,31]. We demonstrated raised ITGB1 proportions in Tet-on HepG2 cells and reduced ITGB1 proportions in LKO mice. Given that ITGB1 was denoted as promotor of EGFR or ERK phosphorylation [13,32,33,34], ITGB1 can impact referred cross-talk between MMP14 and the PRSS8-EGFR-ERK axis.

When concerning the PRSS8 soluble form, little is yet known about post-translational processing that allows this protein to be secreted or its clear global function. Because of this, we examined the association between serum PRSS8 levels and existence of type 2 diabetes mellitus (T2DM) patients, biomarkers of arteriosclerosis, and insulin secretion in human individuals. Max IMT is related to the probability for cardiovascular events [35,36], while HOMA-β is broadly used as estimator of β-cell function [37,38]. The correlation among serum PRSS8 levels and T2DM, and max IMT in T2DM patients suggests that serum PRSS8 may reflect the pathogenesis of T2DM and atherosclerosis. It is conceivable that circulating PRSS8 may directly affect tissues/cells related to insulin secretion and vascular homeostasis. Further studies are needed to clarify pathophysiological significance of circulating PRSS8 in glucose metabolism and diabetes-associated vascular complications.

In summary, our observations further substantiate that PRSS8 overexpression in the liver improved glucose and lipid metabolism dysfunction and fatty liver, by virtue of ERK phosphorylation, which was led to by cross-talking encompassing MMP14 and ITGB1 to the PRSS8-EGFR-ERK axis (Supplementary Figure S6). Correspondingly, the human assessment explicated the serum PRSS8 levels as concomitant major effectors in the development of T2DM, atherosclerosis, and cardiovascular events, and as a putative biomarker for all these evidences might aid in understanding and mediating the regulatory mechanisms of PRSS8 overexpression over glucose and lipid metabolism, as well as excessive hepatic fat storage.

## 4. Materials and Methods

### 4.1. Generation of Liver-Specific PRSS8 Overexpressing Transgenic Mice

For mice transgenesis, a plasmid containing mouse albumin promoter was used, along with the cDNA coding for human PRSS8 (accession code NM-002773, NCBI Reference Sequence Database) that cloned into the Xho I/Sal I and Not I sites of its polylinker. After sequencing verification, the transgene was excised by digestion with EcoR V and Sac II, purified in an agarose gel electrophoresis, and ultimately injected into the pronuclei of fertilized eggs obtained from superovulated female mice (C57BL/6J hybrids). Hence, these injected eggs were surgically transferred to the oviducts of surrogate females. Presence of hPRSS8 transgene in the progeny was confirmed either by PCR amplification, applying hPRSS8 specific primers and mouse tail genomic DNA as a template, or by Southern blot assays using hPRSS8 and polylinker region cut at Pvu II site, as a probe identifying this 1053 bp integrated DNA. The resultant founder mice in the C57BL/6J hybrid background were backcrossed into the original C57BL/6J background for 10 generations, before experimentations, then were labeled as LTg mice. All the procedures above were conducted by UNITECH Co., Ltd. (Chiba, Japan), whereas genotyping was performed by our team through PCR amplification of tail DNA from each mouse, collected at 4 weeks of age. The PCR primers here implied were 5′-CCCACTAGCCTCTGGCAAAC-3′ and 5′-CGCCTTCATAGGTGATGCTG-3′.

### 4.2. Liver-Specific PRSS8 Knockout Mice

LKO mice had been created in a preceding study [1], being employed in the present work together with Flox mice as controls.

### 4.3. Animal Studies

Mice were housed under a 12-h light–dark cycle and provided SD (Oriental Yeast Co., Ltd., Tokyo, Japan) and water ad libitum. At 8 weeks of age, mice were fed SD or a HFD (Research Diets Inc., New Brunswick, NJ, USA) starting. All experiments in this study were conducted using male littermates 23–26 weeks of age after overnight fast (14 h), unless otherwise stated. Every serum level analysis was done by Oriental Yeast Co., Ltd. (Shiga, Japan). Liver triglyceride levels were sent to and measured by Skylight Biotech Co., Ltd. (Akita, Japan).

### 4.4. Metabolic Tests

The GTT and PTT were executed with mice fasted overnight (14 h), then receiving an intraperitoneal injection of glucose (2 g/kg for mice fed SD or 1 g/kg for mice fed HFD) or pyruvate (2 g/kg for mice fed both with SD and HFD). Blood glucose was finally assessed by the ONE TOUCH Verio IQ (Johnson & Johnson, New Brunswick, NJ, USA) immediately before and after the injection at time points of interest.

### 4.5. DNA Microarray

Total RNA was extracted from liver and microarray analysis was performed at Filgen, Inc. (Aichi, Japan) using GeneChip Mouse Gene 2.0 ST Array (Affymetrix, Inc., Santa Clara, CA, USA). The result was analyzed using Affymetrix GeneChip Command Console 4.0, and gene ontology analysis was conducted using DAVID bioinformatics resources 6.8.

### 4.6. Real-Time Polymerase Chain Reaction

Total RNA of tissues (liver tissues and WAT) or cultured cells was prepared by treatment with Trizol reagent (Life Technologies, Carlsbad, CA, USA). Reverse transcription was carried out using Random Primers (Invitrogen, Waltham, MA, USA) and ReverTra Ace (Toyobo Co., Ltd., Osaka, Japan). Quantitative RT-PCR was performed on Quant Studio 7 Flax using FAST SYBR Green Mastermix (Applied Biosystems, Waltham, MA, USA), primer sequences being available upon request. Data were normalized either to the ribosomal protein lateral stalk subunit P0 or to glyceraldehyde 3-phosphate dehydrogenase levels and analyzed applying the comparative CT method. A list of primers was shown in Supplementary Table S2.

### 4.7. Western Blotting

Tissues or cell lysates were prepared by homogenization in the lysis buffer (4M Urea, 150 mM NaCl, 2% Sodium Dodecyl Sulfate, 1 mM Ethylene-Diamine-Tetraacetic Acid and 50 mM Tris-Base). Protein lysates were subjected to Tris-Glycine gel electrophoresis and probed with primary antibodies directed against hPRSS8 (1:1000, BD Transduction Laboratories, Franklin Lakes, NJ, USA), Akt, Akt phosphorylation (Ser473), ITGB1, EGFR, EGFR phosphorylation (Tyr1068), ERK, β-actin (1:1000, Cell Signaling, Danvers, MA, USA), ERK phosphorylation (Thr202/Tyr204) (1:2000, Cell Signaling), MMP14 (1:2000, Abcam, Cambridge, Cambridgeshire, England), and CD36 (1:1000, Novus Biologicals, Centennial, CO, USA). For quantification analysis of the western blot images, ImageJ 1.52 (U. S. National Institutes of Health, Bethesda, MD, USA) was used. The measurement of density profiles was normalized with total protein (in phosphorylation protein analysis) or β-actin (all other proteins).

### 4.8. ELISA

ELISA assays were conducted for measurement of serum fasting insulin levels in WT/LTg mice with a LBIS Mouse Insulin ELISA Kit (AKRIN-011T, FUJIFILM Wako Shibayagi, Gunma, Japan). Measurement of serum fasting PRSS8 levels in WT/LTg mice was used by Human Prostasin ELISA Kit (BOSTER Biological Technology Co., Ltd. Pleasanton, CA, USA).

### 4.9. Histologic Examination and Immunohistochemistry

Livers were removed from mice, fixed in 10% buffered formalin and subsequently embedded in paraffin. Then, 3 μm sections were prepared and stained with hematoxylin and eosin, whereas the remaining sections were deparaffinized and processed for immunohistochemical analysis. Heat-induced epitope retrieval was performed in Target Retrieval Solution, pH 9 (Dako, Santa Clara, CA, USA). Endogenous peroxidases were inactivated using 3% H_2_O_2_, followed by blocking with 3% bovine serum albumin. Serial sections were incubated overnight (4 °C) with anti-CD36 antibody (1:200, Novus Biologicals, Centennial, CO, USA); they were incubated with Envision Dual Link System-HRP (Dako). Finally, they were counterstained with Liquid DAB+ Substrate Chromogen System (Dako) and hematoxylin, the respective images having been captured using an Olympus IX-71 microscope (Olympus Corp., Tokyo, Japan).

### 4.10. In Vitro Studies

HepG2 cells were purchased from ATCC and maintained in Dulbecco’s modified Eagle medium, containing 10% fetal bovine serum. For gene-silencing assays, HepG2 cells were transfected with human PRSS8 small interfering RNA (siRNA) (Silencer Select Validated siRNA, ID: s11274, Ambion, Waltham, MA, USA) or control siRNA (ID: 4390844), resorting to Lipofectamine RNAiMAX (Invitrogen, Waltham, MA, USA) according with manufacturer instructions. After 72 h from transfection, these cells were harvested. Tet-on HepG2 cells were generated by applying Tet-One Inducible Expression System (Takara Bio, Inc. Shiga, Japan), in accordance to the instructions of the manufacturer. Here the cDNA for hPRSS8 (same cDNA as the one mentioned for generation of the LTg mice) was used. Cells were treated with 100 or 1000 ng/mL doxycycline (Dox) for 72 h prior to being harvested.

### 4.11. PRSS8 Activity Quantification

PRSS8 activity of the membrane fraction from Tet-on HepG2 cell lysates was measured following the methods described in a previous study using a fluorescent substrate [26]. Acetyl-Lys-His-Tyr-Arg-α-(4-methylcoumaryl-7-amide), a sequence which is known as target by PRSS8, was synthesized by Peptide Institute Inc. (Osaka, Japan). Thus, the membrane fraction of Tet-on HepG2 cell lysates obtained beforehand were treated with T-PER Tissue Protein Extraction Reagent (Thermo Fisher Scientific, Waltham, MA, USA) and subject to ultracentrifugation. Trypsin as the positive control and T-PER as the negative control were used. Data of protein activity were collected with a SPECTRA MAX GEMINI EM (Molecular Devides, San Jose, CA, USA) at λex = 485 nm and λem = 538 nm. On its turn, MMP14 activity in Tet-on HepG2 cells and WT/LTg mice fed with a HFD was determined by virtue of the SenosoLyte 520 MMP-14 Assay Kit Fluorimetric (AnaSpec, Inc., Fremont, CA, USA), applying the instructions from the manufacturer.

### 4.12. Human Studies

In order to assess PRSS8 expression and glucose metabolism in humans, a total of 40 non-DM participants who visited Isawa Hot Spring Hospital (Yamanashi, Japan), for annual health checkup, and a total of 39 T2DM patients who visited the University of Yamanashi Hospital (Yamanashi, Japan), were recruited. Detailed characteristics were shown in Supplementary Table S1. Serum PRSS8 levels in all participants were determined using the same ELISA Kit anteriorly referred for measurement of in vivo serum fasting PRSS8 levels. Moreover, max IMT was registered through carotid ultrasonography only in T2DM patients. HOMA-β is calculated here using the formula that follows: HOMA-β (%) = (fasting immunoreactive insulin [µU/mL] × 360)/(fasting plasma glucose [mg/dL] − 63). Body mass index (BMI) is calculated here using the formula that follows: BMI (kg/m^2^) = body weight (kg)/height (m)^2^. Serum immunoreactive insulin, plasma glucose, LDL- and HDL-cholesterol, and triglyceride levels were measured in SRL, Inc., (Tokyo, Japan).

### 4.13. Statistical Analysis

Data are presented as means ± SEM or median [95% CI]. Statistical significance was determined using either Student’s *t* tests, A two-way repeated measures analysis of variances, Pearson’s product-moment correlation coefficients, or Fisher’s exact tests, as appropriate and later mentioned for each case. Statistical significance was set at 0.05, and all statistical tests were two-tailed. All of these analyses were conducted in EZR v1.41 (Saitama Medical Center, Jichi Medical University, Saitama, Japan), which is a graphical user interface for R (The R Foundation for Statistical Computing, Vienna, Austria). More precisely, it is a modified version of the R commander, designed to add statistical functions frequently used in biostatistics [39].

## Data Availability

The data that support the findings of this study are available from the corresponding author upon reasonable request.

## Supplementary Materials

The following are available online at https://www.mdpi.com/article/10.3390/ijms22158314/s1, Supplementary Figure S1: Serum lipid profile, transaminases and lactate dehydrogenase levels in SD-fed WT and LTg mice, Supplementary Figure S2: CD36 expression in liver of HFD-fed WT and LTg mice, Supplementary Figure S3: Gene and CD36 expression in Tet-on HepG2 cells, Supplementary Figure S4: TLR4 expression in liver of HFD-fed WT and LTg mice and in Tet-on HepG2 cells, Supplementary Figure S5: Serum PRSS8 levels and gene expression in white adipose tissue of HFD-fed WT and LTg mice, Supplementary Table S1: Characteristics of human study participants, Supplementary Table S2: List of primers.
