# Supplementary material for: Liver-Specific Overexpression of Prostasin Attenuates High-Fat Diet-Induced Metabolic Dysregulation in Mice
Source: Int J Mol Sci. 2021 Aug 2;22(15):8314. doi: 10.3390/ijms22158314 (PMC8348244; doi:10.3390/ijms22158314)
Supplement: Supplementary file 1 [file ijms-22-08314-s001.zip › ijms-1243357-supplementary.pdf]

SUPPLEMENTARY MATERIALS

Liver-Specific Overexpression of Prostasin Improves High-Fat Diet-Induced Metabolic Dysregulation in Mice

Tetsuo Sekine, Soichi Takizawa, Kohei Uchimura, Asako Miyazaki and Kyoichiro Tsuchiya

SUPPLEMENTARY FIGURE

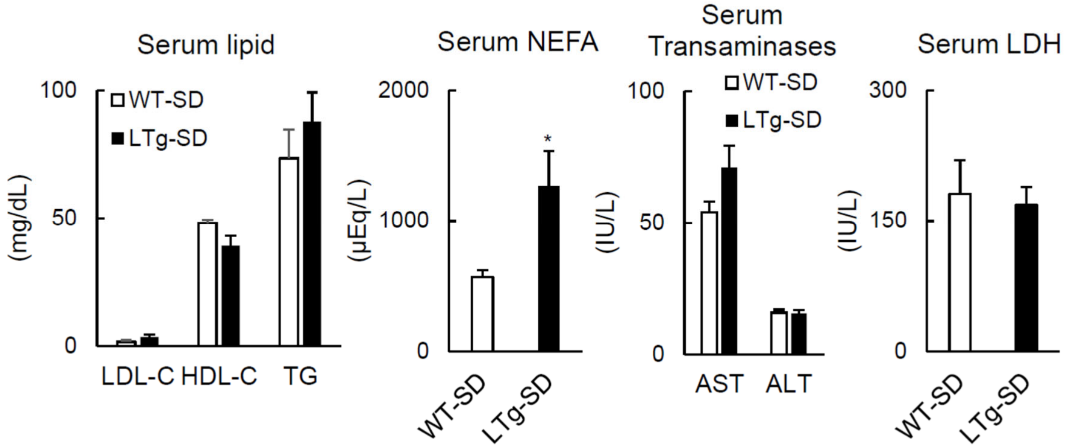

Supplementary Figure S1. Serum lipid profile, transaminases and lactate dehydrogenase levels in SD-fed WT/LTg mice. Serum low- and high-density lipoprotein cholesterol (LDL-C and HDL-C), triglycerides (TG), non-esterified fatty acid (NEFA), aspartate (AST) and alanine aminotransferase (ALT), and lactate dehydrogenase (LDH) levels in standard diet (SD)-fed WT/LTg mice under overnight fasting (14h) (n = 4 mice per group). Values are shown as the mean ± SEM; * p-value < 0.05 (t test).

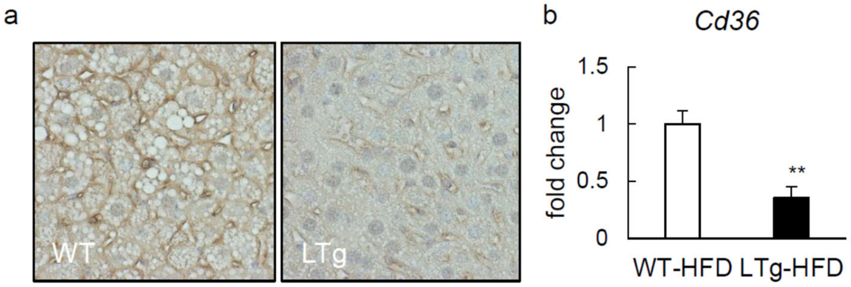

Supplementary Figure S2. CD36 expression in liver of HFD-fed WT/LTg mice. (a) Representative pictures of cluster of differentiation (CD) 36 immunostainings in liver of HFD-fed WT/LTg mice (x400). (b) *Cd36* gene expression in liver of HFD-fed WT/LTg mice under overnight fasting (14h) (n = 6–7 per group). Values are shown as the mean ± SEM; ** *p*-value < 0.01 (*t* test).

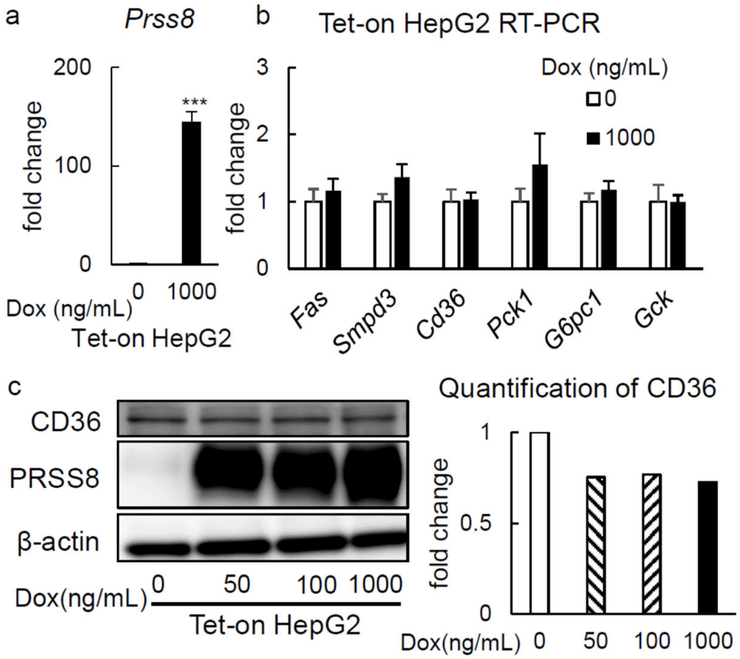

Supplementary Figure S3. Gene and CD36 expression in Tet-on HepG2 cells. (a) *Prss8* and (b) other various gene expression in liver of the Tet-on HepG2 cells under 72 hours exposure to each concentration of doxycycline (Dox) (n = 5–6 per group). Values are shown as the mean ± SEM; * *p-value* < 0.05, ** *p-value* < 0.01, and *** *p-value*

< 0.001 (t test). (c) Representative western blotting analysis and CD36 quantification using densitometry of three independent experiments in the Tet-on HepG2 cells under 72 hours exposure to each concentration of Dox.

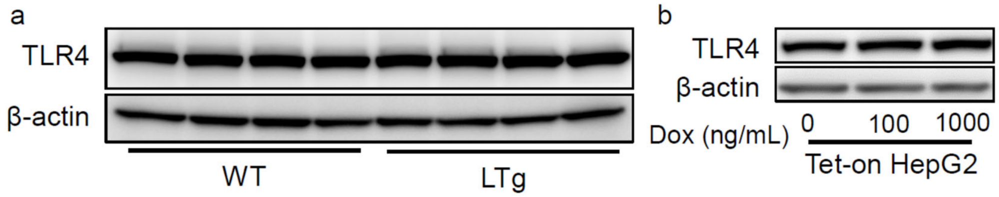

Supplementary Figure S4. TLR4 expression in liver of HFD-fed WT/LTg mice and in Tet-on HepG2 cells. (a) Western blotting of toll-like receptor (TLR) 4 in liver of HFD-fed WT/LTg mice under overnight fasting (14h) (n = 4 per group). (b) Western blotting of TLR4 in the Tet-on HepG2 cells under 72 hours exposure to each concentration of doxycycline (Dox).

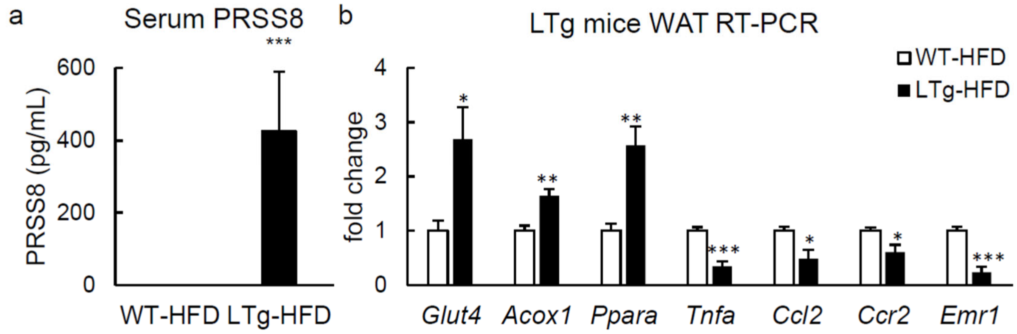

Supplementary Figure S5. Serum PRSS8 levels and gene expression in white adipose tissue of HFD-fed WT/LTg mice. (a) Serum hPRSS8 levels in HFD-fed WT/LTg mice under overnight fasting (14h) (n = 4 per group). (b) Gene expression levels in the epididymal white adipose tissue (WAT) of HFD-fed WT/LTg mice under overnight fasting (14h) (n = 5–7 per group). Values are shown as the mean ± SEM; * p-value < 0.05, ** p-value < 0.01, and *** p-value < 0.001 (t test).

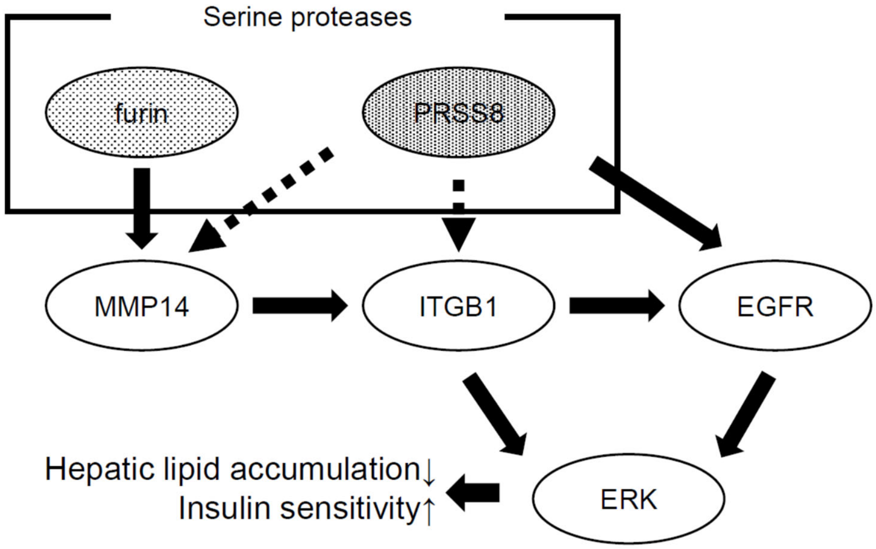

Supplementary Figure S6. The putative signaling pathways involved with a hypothetic schema. PRSS8; Prostasin, MMP14; Matrix metalloproteinase 14, ITGB1; Integrin subunit β1, EGFR; Epidermal growth factor receptor, ERK; Extracellular signal-regulated kinase

Solid lines; Pathways shown in the previous studies
Dotted lines; Putative pathways shown in the present study
Furin, one of the serine proteases, cleavages and activates MMP14. MMP14 activates ITGB1. ITGB1 promotes ERK phosphorylation (directly or via EGFR phosphorylation). The present study showed that the PRSS8 could promote ERK phosphorylation via activating MMP14 and ITGB1 and promoting EGFR phosphorylation. ERK phosphorylation reduces hepatic lipid accumulation and improves insulin sensitivity.

Supplementary Table S1. Characteristics of human study participants

	Non-DM	T2DM	p-value
Sex (male, n [%])	24 (60.0)	21 (53.8)	0.65
Age, median [95% CI] (years)	62 [48–72]	68 [45–78]	0.07
BMI, median [95% CI] (kg/m^2)	22.1 [18.1–27.2]	24.7 [18.7–35.4]	<0.01
Blood pressure, median [95% CI] (mmHg)			
Systole	117 [102–128]	134 [108–163]	<0.0001
Diastole	74 [58–85]	70 [59–88]	0.64
FPG, median [95% CI] (mg/dL)	95 [82–110]	161 [109–283]	<0.0001
#IRI, median [95% CI] (μU/mL)	2.86 [1.02–5.06]	6.40 [1.47–19.8]	<0.0001
Lipid profile, median [95% CI] (mg/dL)			
LDL-C	119 [79–160]	123 [73–210]	0.16
HDL-C	60 [43–87]	44 [33–70]	<0.0001
TG	73 [41–232]	131 [59–321]	<0.0001
Smoking, n (%)	15 (37.5)	19 (48.7)	0.37
Medication			
OHA, median [95% CI] (number of agents)	NA	1 [0–4]	NA
Insulin / GLP-1 RA, n (%)	NA	12 (30.8)	NA

DM; diabetes mellitus, BMI; body mass index, FPG; fasting plasma glucose, IRI; immunoreactive insulin, LDL-C; low-density lipoprotein, HDL-C; high-density lipoprotein, TG; triglyceride, OHA; oral hypoglycemic agent, GLP-1RA; glucagon-like peptide-1 receptor agonist, NA; not applicable

Statistical analysis; Fisher's exact test (age and smoking), t test (all others)

[#]T2DM patients with insulin therapy were excluded.

Supplementary Table S2. List of primers

mRplp0	Fw	ACCGCCTGGTTCTCCTATAA
	Rv	AAGACGATGTCACTCCAACG
mGapdh	Fw	TCAGGAGAGAGTGTTTCCTCGT
	Rv	GCAACAATCTCCACTTTGCC
mCidea	Fw	CAGAAATGGACACCGGGTAG
	Rv	CTCGTACATCGTGGCTTTGA
mCidec	Fw	GGTATTGCCAGGAGGCTG
	Rv	CTGCCACATGCCTGGAC
mFgf21	Fw	AGCCAGGGGTCATTCAAATC
	Rv	CAGGATCAAAGTGAGGGGAT
mIl1b	Fw	GCCACCTTTTGACAGTGATG
	Rv	CCCAGGTCAAAGGTTTGGAA
mTgfb	Fw	CCTGAGTGGCTGTCTTTGA
	Rv	GGGCTGATCCCGTTGATTTC
mTnfa	Fw	ACCCTCACACTCAGATCATCTTC
	Rv	TGGTGGTTTGCTACGACGT
mCol1a1	Fw	CGCTGGTGCTGCTGAC
	Rv	TTCTCCTTTCTGCCCCTTTG

mCol1a2	Fw	TTGCAATCGGGATCAGTACGA
	Rv	CACGTGGTCCTCTGTCTCCAG
mMmp14	Fw	AGAGAACTTCGTGTTGCCTG
	Rv	TGACCCTGACTTGCTTCCATA
mTimp2	Fw	AAGGAGTATCTAATTGCAGGAAAGG
	Rv	GCTCTTCTTCTGGGTGATGC
mCd36	Fw	GCCAAGCTATTGCGACATGA
	Rv	ACAGCGTAGATAGACCTGCAA
mGlut4	Fw	CCGGACCCTATACCCTATTCA
	Rv	GGGTTCCCCATCGTCAGAG
mAcox1	Fw	TCATGTGGTTTAAAAAACTCTGTGC
	Rv	CGTGATCTCCAGATTCCAGG
mPpara	Fw	GAACTTAGAGGAGAGCCAAGTT
	Rv	GACCATGTTGGATGGATGTG
mCcl2	Fw	ATCCCAATGAGTAGGCTGGA
	Rv	GAGCTTGGTGACAAAAACTACA
mCcr2	Fw	ACAAATCAAAGGAAATGGAAGAC
	Rv	TGCCGTGGATGAACTGAGG
mEmr1	Fw	CTTTGGCTATGGGCTTCCAGTC
	Rv	GCAAGGAGGACAGAGTTTATCGT
hRplp0	Fw	GCAAGAACACCATGATGCG
	Rv	AGCAGCTGGCACCTTATTG
hMmp14	Fw	CGTCCATCAACACTGCCTAC
	Rv	ACCCAATGCTTGTCTCCTTTG
hTimp2	Fw	ACGACATTTATGGCAACCCT

	Rv	CAGGCCCTTTGAACATCTTTATC
hPprss8	Fw	CCAGCTAGACTCCTACTCCG
	Rv	GAGTTGGAGGAGTGCAATGT
hFas	Fw	ATGAGGCTGTGAAGCCATTC
	Rv	GGTCTATGAGGCCTATCTGGA
hSmpd3	Fw	GATAACTGCTCCTCTGACGAC
	Rv	GCACACATCCTCATCGTACA
hCd36	Fw	GGTCCTTATACGTACAGAGTTCG
	Rv	GTTGTCAGCCTCTGTTCCAA
hPepck1	Fw	ATCGTCACCCAAGAGCAAAG
	Rv	ACATGGTGCGACCTTTCAT
hG6pase	Fw	CGTGATGGTCACATCTACTCTT
	Rv	TGACATTCAAGCACCGAAATC
hGck	Fw	GCGGAGAAGCCTTGGATATT
	Rv	GGATCTGCTCTACCTTCTCCT
